# Overexpression of the Maize *psbA* Gene Enhances Drought Tolerance Through Regulating Antioxidant System, Photosynthetic Capability, and Stress Defense Gene Expression in Tobacco

**DOI:** 10.3389/fpls.2015.01223

**Published:** 2016-01-12

**Authors:** Yongjin Huo, Meiping Wang, Yangyang Wei, Zongliang Xia

**Affiliations:** ^1^State Key Laboratory of Wheat, Maize Crop Science in Henan ProvinceSynergetic Innovation Center of Henan Grain; ^2^Crops, College of Life Science, Henan Agricultural UniversityZhengzhou, China

**Keywords:** antioxidant enzyme, psbA, drought, photosynthesis, tobacco

## Abstract

The *psbA* (encoding D1 protein) plays an important role in protecting photosystem II (PSII) from oxidative damage in higher plants. In our previous study, the role of the *psbA* from maize (*Zea mays*. L) in response to SO_2_ stress was characterized. To date, information about the involvement of the *psbA* gene in drought response is scarce. Here we found that overexpression (OE) of *ZmpsbA* showed increased D1 protein abundance and enhanced drought stress tolerance in tobacco. The drought-tolerant phenotypes of the OE lines were accompanied by increases of key antioxidant enzymes SOD, CAT, and POD activities, but decreases of hydrogen peroxide, malondialdehyde, and ion leakage. Further investigation showed that the OE plants had much less reductions than the wild-type in the net photosynthesis rate (Pn), stomatal conductance (Gs), and the maximal photochemical efficiency of PSII (Fv/Fm) during drought stress; indicating that OE of *ZmpsbA* may alleviate photosynthesis inhibition during drought. qRT-PCR analysis revealed that there was significantly increased expression of *NtLEA5, NtERD10C, NtAREB*, and *NtCDPK2* in *ZmpsbA*-OE lines. Together, our results indicate that *ZmpsbA* improves drought tolerance in tobacco possibly by alleviating photosynthesis reduction, reducing reactive oxygen species accumulation and membrane damage, and modulating stress defense gene expression. *ZmpsbA* could be exploited for engineering drought-tolerant plants in molecular breeding of crops.

## Introduction

Photosystem II consists of a multi-protein complex and plays important roles in the oxygen-evolving photosynthetic organisms (Wollman et al., 1999). In PSII reaction center, D1 and D2 proteins can together bind most of the redox-active cofactors and participate in electron transfer activities (Nelson and Yocum, 2006).

The D1 protein (encoded by the *psbA* transcripts) is an indispensable component of oxygenic photosynthesis in higher plants. In *Arabidopsis*, tobacco and maize, the *psbA* gene exists in a single copy in the chloroplast genomes (Mulo et al., 2012). To date, studies on the function of plant *psbA* mainly focused on the model plant *Arabidopsis* (Adir et al., 2003). The D1 protein is prone to constant photo damage because of strong oxidative chemistry of PSII water splitting. Through the PSII repair machinery, damaged D1 protein will be degraded and replaced by a new one so as to maintain stable photosynthesis (Baena-González and Aro, 2002; Adir et al., 2003). Increasing molecular and genetic evidence has revealed that the proteases FtsH and Deg are responsible for D1 degradation, and the proteases FtsH2 and FtsH5 play important roles in the D1 repair cycle in *Arabidopsis* (Maxwell and Johnson, 2000; Müller et al., 2001). Armbruster et al. (2010) found a thylakoid protein PAM68, which is necessary for D1 biogenesis and assembly of PS II complex (Armbruster et al., 2010). Subsequently, Zhang et al. (2011) reported that a chloroplast-localized protein HCF243 functioned in maintaining D1 stability in *Arabidopsis* (Zhang et al., 2011). A more recent report showed that the D1 protein had a C-terminal processing, which is also necessary for PSII function in *Arabidopsis* (Che et al., 2013). In addition, it has been reported that the D1 protein of PSII was protected from oxidative damage and degradation in the drought-tolerant transgenic plants (Almoguera et al., 2012).

Although new insights in D1 protein function were produced in model plants, the knowledge of D1 function and regulation from crops is very limited. As an important cereal and forage crop, maize often suffers for drought stress. Thus, drought has already become a serious problem for maize production due to climate change.

Unfortunately, the molecular aspects of D1 responsive to drought in maize are largely unclear. Previously, a putative SO_2_ stress-induced mRNA encoding the D1 protein from *Zea m*ays (*ZmpsbA*) was isolated by mRNA differential display, and its involvement of SO_2_ stress response was characterized (Su et al., 2015). To date, information about the involvement of the maize *psbA* gene in drought response is scarce. Here, we further investigate its drought tolerance and possible function mechanisms in transgenic tobacco.

## Materials and Methods

### Plant Materials, Growth Conditions, and Stress Treatments

The sense *ZmpsbA* transgenic tobacco (*Nicotiana tabacum*cv. Xanthi) lines (OE-8 and OE-11) and wild type (WT) were used in this study. *ZmpsbA*, a cDNA sequence encoding D1 protein (accession number AF543684), was isolated from maize (*Zea m*ays L.) by mRNA differential display reverse transcription-polymerase chain reaction (DDRT-PCR). The 35S:*ZmpsbA* transgenic plants were produced in our lab (Su et al., 2015). The sense transgenic plants were T_3_ homozygous generation. The tobacco seeds were surface sterilized and then germinated on plates containing MS medium. After one week, the seedlings were transferred to sterilized low-nutrient soil to obtain fully grown plants. Tobacco plants were grown in a growth room at approximately 25°C, 60–70% relative humidity, and a photoperiod of 16-h-light/8-h-dark and light intensity of 200 μmol m^-2^ s^-1^, as described before (Xia et al., 2013).

For stress treatments, 3-week-old seedlings from both transgenic lines and WT were treated with 20% of PEG8000 and 250 mM NaCl, respectively, for drought or salt stress. For high temperature stress, the whole plants were exposed to 42°C in the chamber under the light. For high light stress, the same age plants were exposed to a light intensity of 1200 μmol m^-2^ s^-1^. Meanwhile, another group of plants was treated under normal conditions as controls. After treatments, leaf samples were collected at indicated time and used for D1 protein expression analyses.

### Analysis of *ZmpsbA* OE Lines for Drought Stress Tolerance

Wild-type and both transgenic lines (OE-8 and OE-11) were grown in MS medium for one week, and then these seedlings were transplanted into small pots with soil (four seedlings for each pot, and three pots for each transgenic line), and cultured for another two weeks under normal conditions. These 3 weeks old plants were subjected to progressive drought by withholding water for about 11 days when wild-type plants showed close to be lethal. After treatments, the fresh weight per plant and remaining chlorophyll content of the WT and OE lines were determined. The stress assay was conducted three times, which produced similar results.

### Detection of D1 Protein by Immunoblotting

Thylakoid membranes from leaf samples were prepared as described previously (Zhang et al., 1999). The tobacco leaves were homogenized in the extraction buffer, containing 50 mM HEPES, pH 7.5, 10 mM NaF, 5 mM MgCl_2_, and filtered through 3∼4 layers of gauze. The filtrate was centrifuged at 5000 × *g* for 5 min. The thylakoid pellets were washed and centrifuged again, and finally suspended in the extraction solution for use. The thylakoid membrane protein concentration was determined as described by Bradford (1976). 20 μg of membrane protein were fractionated and transferred onto a nitrocellulose membrane, which was thereafter blotted with the D1-specific polyclonal antibody raised in rabbits (Agrisera). Finally, the blotted signals were detected with a horseradish peroxidase-conjugated goat anti-rabbit IgG secondary antibody (Agrisera) using the 3, 3′-diaminobenzidine (DAB) development kit (Bio Basic Inc., Canada). Quantitative evaluation of the signals was done using the Tanon GIS system (Tanon, Shanghai, China).

### Determination of H_2_O_2_, MDA Content, and Ion Leakage (IL)

H_2_O_2_ content was assayed according to our previously used method (Xia et al., 2012). The absorbance of the resulting solution was measured at 415 nm and the H_2_O_2_ concentration was determined using a standard curve plotted with standard concentrations of H_2_O_2_ (Ferguson et al., 1983). MDA content was determined as described previously (Draper and Hadley, 1990; Xia et al., 2014). Ion leakage (IL) was measured according to the method of Zhou et al. (2012). In these experiments, three independent biological replications were conducted, and three times were done in each independent assay.

### Measurements of SOD, CAT, and POD Activities

The activities of SOD, CAT, and POD were spectrophotometrically measured. Total SOD activity was determined as reported by us previously (Liu et al., 2013). CAT activity was determined according to the method used by us (Xia et al., 2012), and POD activity was determined by following the method described by Cakmak and Horst (1991).

### Measurements of Photosynthetic Gas Exchange and Chlorophyll Fluorescence Parameters

The LI-6400 portable photosynthesis analyzer (LI-COR, USA) was used to measure the net photosynthetic rate (*Pn*), stomatal conductance (*Gs*) and intercellular CO_2_ concentration (*Ci*). The measurements were performed under the following conditions: 800 μmol m^-2^ s^-1^ PFD, 500 μmol s^-1^ flow rate, leaf temperature 30 ± 2°C, and relative humidity 60∼70%. Before measure, plants were kept at 100 μmol m^-2^ s^-1^ PFD for 1 h to maintain stomatal opening, and then transferred to stay at 800 μmol m^-2^ s^-1^ PFD for 20 min to be acclimated.

Chlorophyll fluorescence was measured using a pulse-modulated fluorimeter (FMS-2, Hansatech, UK) on tobacco leaves as described previously (Genty et al., 1989). The maximum PSII quantum yield (*Fv/Fm*) was determined in dark-adapted (15 min) leaves. After the initial chlorophyll fluorescence yield (*Fo*) was determined in low modulated measuring light, a 0.8 s pulse of saturating white light (6000 μmol m^-2^ s^-1^) was applied to obtain the maximum chlorophyll fluorescence yield (*Fm*′) and the *Fv/Fm* (*Fv*, the variable chlorophyll fluorescence yield, is defined as *Fm*–*Fo*). The steady-state fluorescence level (*Fs*) and the maximum chlorophyll fluorescence level (*Fm*) during exposure to 800 μmol m^-2^ s^-1^ illumination were also measured. The actual quantum yield of PSII electron transport (*Φ_PSII_*) was calculated as (*Fm*′–*Fs*′)/*Fm*′.

### Quantitative Real-time PCR

Quantitative real-time PCR was used to determine transcript levels of *ZmpsbA, NtLEA5, NtERD10C, NtAREB*, and *NtCDPK2*. The qRT-PCR was performed on an IQ5 light cycler system (Bio-Rad) using SYBR Premix (Thermo Scientific, USA) with gene-specific primers (Supplementary Table S1). The tobacco *Actin2* transcript was used as an internal control (Xia et al., 2013) and the transcript levels of genes were calculated according to the 2^-ΔΔC^_T_ method (Livaka and Schmittgen, 2001). All qRT-PCR experiments were performed with three biological and three technical replicates.

## Results

### Abundance of *ZmpsbA* (Encoding D1) in Transgenic Plants

**FIGURE 1:**
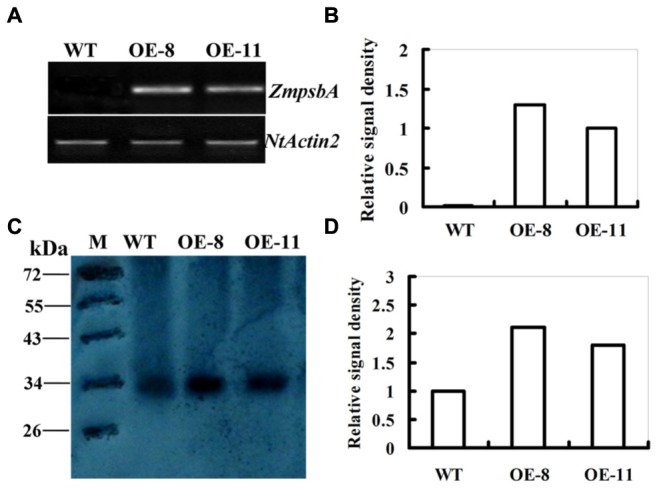
**Expression levels of *ZmpsbA* in transgenic tobacco plants. (A)** Transcription levels of the *ZmpsbA* in wild-type and both over-expression (OE) lines (OE-8 and OE-11). *ZmpsbA* transcripts detected by semi-quantitative RT-PCR were present in the OE lines, but not in the wild-type plants. The tobacco *Actin2* transcript was used as a control for equal cDNA amounts. **(B)** Relative signal density of *ZmpsbA* gene expression in transgenic tobacco. **(C)** Western blot analysis of D1 protein abundance in wild-type and both OE lines (OE-3 and OE-7). Proteins (20 μg per lane) were fractionated by 12.5% SDS-PAGE and immunobloted with D1 protein antibody. **(D)** Relative signal density of D1protein (32 kDa) in WT and OE lines.

In our previous study, six homozygous lines harboring *35S:ZmpsbA* constuct were generated. Among the lines OE-8 and OE-11 had higher *ZmpsbA* expression levels detected by qRT-PCR. Due to the high similarity in nucleotide sequences (91%) between maize and tobacco, *psbA* was also detected in the WT (Su et al., 2015). To exclude the disturbance of endogenous *psbA* gene in transgenes, a new specific pair of primers was designed for confirming *ZmpsbA* expression in both OE lines, according to the *psbA* sequence alignments between maize and tobacco (**Supplementary Figure S1**). Semi-quantitative RT-PCR was conducted using *ZmpsbA*-specific primers (ZmpsbA- sqF1 and ZmpsbA-sqR1, Supplementary Table S1). A 275-bp PCR product was amplified from both transgenic lines (OE-8 and OE-11), while no product was amplified from the WT plants (**Figure 1A**). Moreover, the transcript level of the *ZmpsbA* was relatively high in OE-8 compared with that in OE-11 (**Figure 1B**). This result indicates that the *ZmpsbA*-overexpression (OE) lines were obtained.

Analysis of D1 abundance by protein gel blot showed that the level of D1 protein (about 32 kDa) in transgenic lines was higher than that in the WT plants (**Figure 1C**). In both OE-8 and OE-11, D1 protein abundance was 1.1 and 0.8-fold higher than that of the WT, respectively (**Figure 1D**). This indicates that over-expression of *ZmpsbA* markedly increased the level of D1 protein under normal growth conditions.

### Responses of D1 Protein Abundance to Drought, Heat, Salt, or High Light Stress in *ZmpsbA* OE lines

**FIGURE 2:**
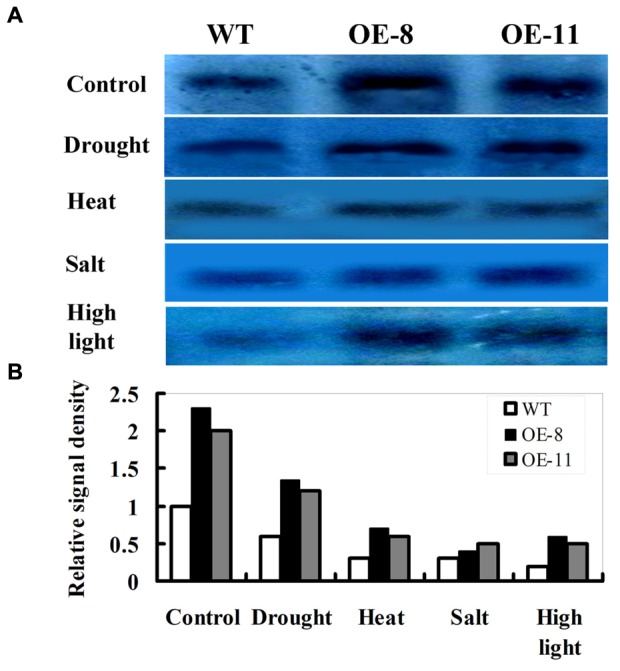
**Responses of D1 protein abundance to drought, heat, salt, or high light stress in *ZmpsbA* OE lines by immunoblot analysis. (A)** D1 protein abundance in 3-week-old WT and OE plants under drought (20% PEG-8000 for 12 h), heat (42°C for 3 h), salt (250 mM NaCl for 12 h) or high light (1200 μmol m^-2^ s^-1^ for 1 h) conditions. **(B)** Relative signal density of D1protein abundance in WT and OE lines under above stress conditions.

In immunoblot experiments, tobacco leaves from lines OE-8 and OE-11 were used to examine the effects of drought, heat, salt, or high light stress on D1 protein abundance. As shown in **Figure 2A**, D1 protein was detected under normal or stress conditions. Under normal conditions, the levels of D1 protein in transgenic plants (OE-8 and OE-11) were much higher than that in WT because of the over-expression of *ZmpsbA*. Following drought (20% PEG for 12 h), heat (42°C for 3 h), salt (250 mM NaCl for 12 h) or high light (1200 μmol m^-2^ s^-1^ for 1 h), D1 protein abundance was markedly decreased in both transgenic plants and WT, but the magnitudes of decreases were differential between WT and OE lines (**Figures 2A,B**). As shown in **Figures 2A,B**, after heat, salt or high light stress, D1 protein abundance was greatly decreased in both WT and OE plants (more than 70% decreases on average). Noticeably, after drought stress, the D1 protein abundance showed less decreases in both transgenic and WT plants (about 40% decreases on average); moreover, the OE lines maintained higher D1 protein levels than the WT, indicating that levels of D1 protein in transgenic plants might be more stable than that in WT during drought stress.

### Performance of *ZmpsbA*-Overexpressing Tobacco Plants to Drought Stress

**FIGURE 3:**
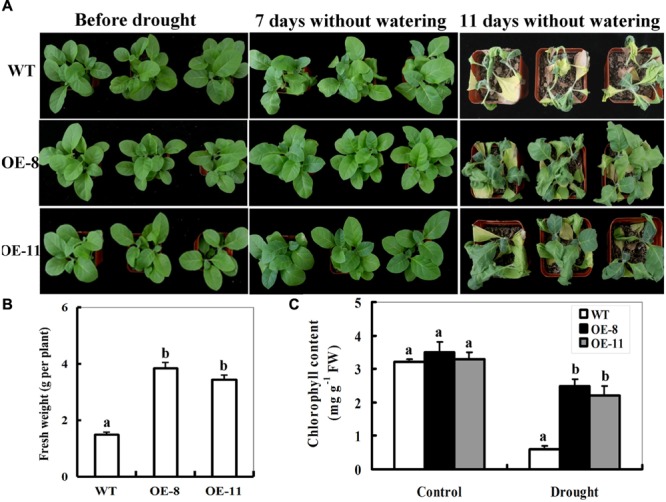
**Phenotypes of wild-type and *ZmpsbA*-transgenic tobaccoplants in response to drought stress. (A)** Drought tolerance of potted plants of wild-type and *ZmpsbA*-OE tobacco. Four-week-old WTand transgenic OE (OE-8 and OE-11) plants were grown in soil in pots for 11 day without watering. **(B)** Fresh weight of 11-day-drought-stressed wild-type and *ZmpsbA*-OE plants. Values are mean ± SE, *n* = 12. **(C)** Chlorophyll content in 11-day-drought-stressed wild-type and *ZmpsbA*-OE plants. Values are mean ± SE, *n* = 12. In both **(B,C)**, statistical analysis was performed using ANOVA test (*P* < 0.05) and significant differences between wild-type and OE lines are indicated by different letters.

To characterize the performance of *ZmpsbA* OE lines under drought stress in soil, both OE lines (OE-8 and OE-11) were tested at the seedling stage (4-week old). Under well-watered conditions, there was no obvious difference between WT and transgenic lines in leaves size and number of plants (**Figure 3A**; left panel). After 7 days without watering, the WT plants displayed wilted (more than 50 % leaves of the wild type plants began to turn soft and rolled), but the leaves of both OE lines had no significant changes (**Figure 3A**; middle panel). After 11 days without watering, all WT plants displayed severe wilting (all leaves were severely curled and more than 80% leaves were turning yellow and dead), whereas *ZmpsbA* transgenic lines showed signs of moderate water stress and most upper leaves of transgenic plants were still green and fully expanded (**Figure 3A**; right panel). Accordingly, plant biomass and remaining chlorophyll content in *ZmpsbA* transgenic lines were significantly higher than those in WT plants (1.5 and 2.8-fold on average, respectively) (**Figures 3B,C**). Three days after re-watering, more than 70% of the WT plants were dead, whereas all transgenic lines survived the stress and started to grow (Data not shown). These results provide evidence that over-expression of *ZmpsbA* in transgenic tobacco plants improves tolerance to drought stress.

### Overexpression of *ZmpsbA* Decreases MDA and H_2_O_2_ Accumulations, and IL Under Drought Stress

**FIGURE 4:**
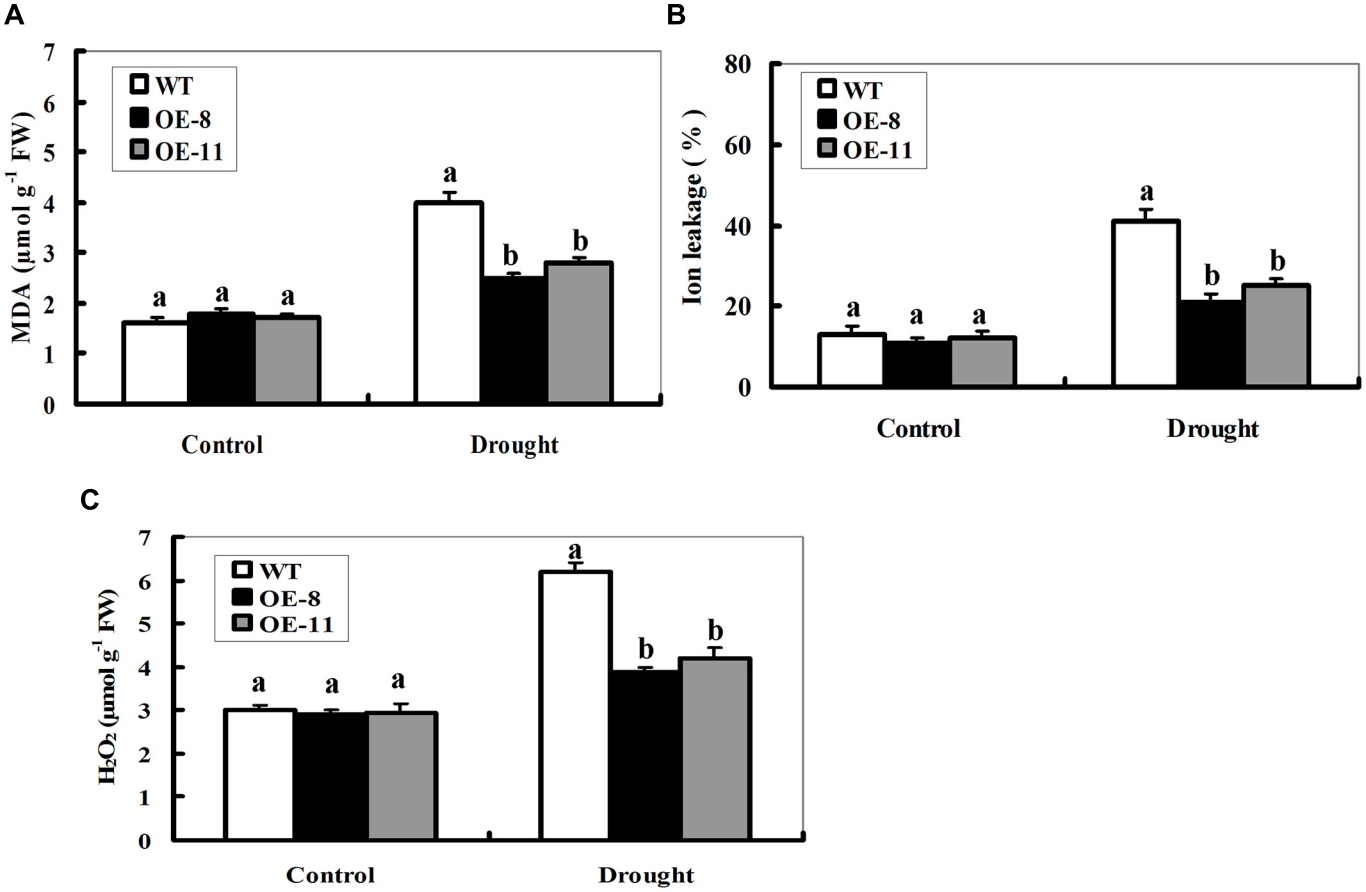
**Changes of MDA, lon leakage, and H_2_O_2_ in *ZmpsbA*-transgenic lines under drought stress. (A)** Determination of MDA accumulation in leaves of WT and both OE lines (OE-8 and OE-11) after 7-day drought stress. **(B)** Determination of ion leakage (IL) in leaves of WT and both OE lines (OE-8 and OE-11) after 7-day drought stress. **(C)** Quantitative determination of H_2_O_2_ accumulation in leaves of WT andboth OE lines (OE-8 and OE-11) after 7-day drought stress. In **(A–C)**, each experiment was repeated three times. Bar indicates SE. Different letters indicate significant differences between wild-type and OE lines (ANOVA; *P* < 0.05).

Enhanced drought tolerance in both *ZmpsbA* transgenic lines prompted us to detect the differences in lipid peroxidation. Malondialdehyde (MDA), a product of lipid peroxidation was measured between the WT and OE plants after 7-day drought treatment. The MDA content was significantly higher in the WT (150% increase) than that in both transgenic lines (39% increase for OE-8 and 65% increase for OE-11), suggesting that the transgenic plants suffered less membrane damage than the wild type (**Figure 4A**). Then, IL measurement showed that transgenic lines had less IL than WT under drought stress (**Figure 4B**), suggesting that transgenic lines were subjected to less membrane injury.

The *ZmpsbA* transgenic plants had lower MDA levels and less IL than WT under drought stress, implying that they may be subjected to less serious oxidative damage than the WT. Therefore, it was of interest to detect reactive oxygen species (ROS) accumulation in the WT and OE lines during drought stress. Quantitative determination of H_2_O_2_ accumulation was performed in 7-days drought-stressed leaves along with controls from OE and WT plants. As shown in **Figure 4C**, H_2_O_2_ content increased in both WT and transgenic lines after drought stress. However, transgenic lines accumulated lower levels of H_2_O_2_ (only 38% increase on average) relative to WT (107% increase) after drought stress (**Figure 4C**). No significant differences in MDA, H_2_O_2_, or IL were observed between WT and both OE lines under control conditions (**Figures 4A–C**). These physiological indices demonstrated that lower ROS accumulation and lipid peroxidation in the transgenic lines may be correlated to their improved tolerance to drought stress.

### Overexpression of *ZmpsbA* Increases Antioxidant Enzyme Activities Under Drought Stress

**FIGURE 5:**
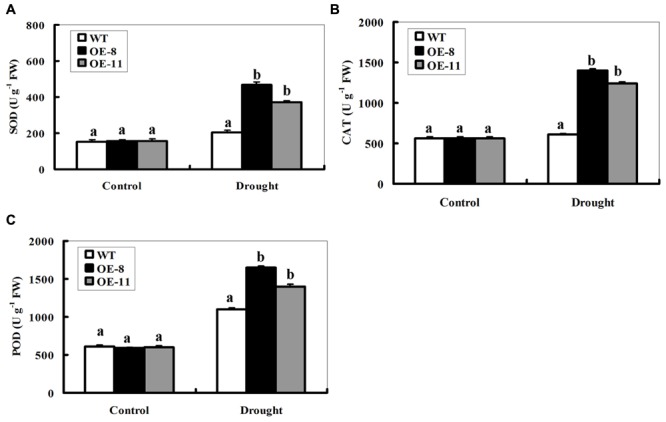
**Activities of antioxidant enzymes SOD, CAT and POD in WT and *ZmpsbA*- transgenic lines.** Four weeks old WTand transgenic plants were drought-stressed for 7 days, and then the leaf samples were taken and used to detect activities of SOD, CAT, and POD. **(A)** SOD activity; **(B)** CAT activity; **(C)** POD activity. Data are means ± SE calculated from three replicates. Different letters indicate a significant difference between the WT and both transgenic lines (ANOVA; *P* < 0.05). Three independent experiments were performed, which produced similar results.

Enzymatic antioxidants play significant roles in ROS homeostasis regulation. Therefore, the activities of three significant antioxidant enzymes, SOD, CAT, and POD were measured in the leaves from potted WT and transgenic plants. Under control conditions, SOD, CAT, or POD activities displayed no difference between transgenic lines and the WT (**Figures 5A–C**). After 7-day drought stress, however, both transgenic lines had significantly higher SOD, CAT, or POD activities than the WT (**Figures 5A–C**). These results, together with ROS levels difference, imply that OE of *ZmpsbA* reduced ROS accumulation by enhancing major antioxidant enzyme activities under drought stress.

### Over-Expression of *ZmpsbA* Improves Photosynthesis Capability Under Drought Stress

**FIGURE 6:**
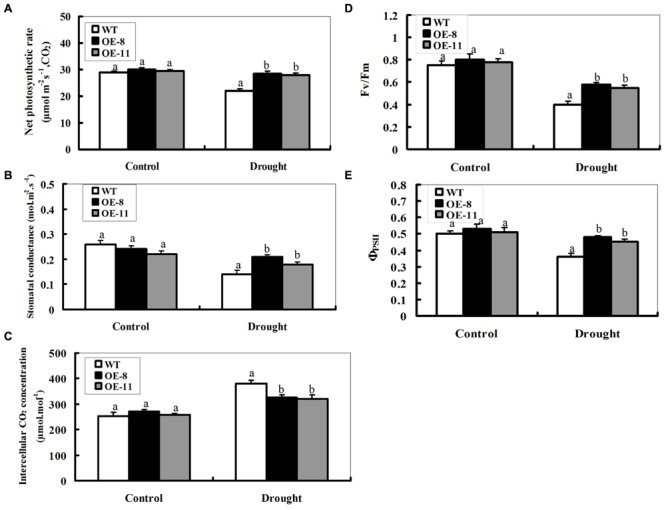
**Photosynthetic performance of the wild-type and *ZmpsbA*-transgenic plants under drought stress.** Four weeks old WT and transgenic plants were drought-stressed for 7 days, and then the leaf samples were taken to determine photosynthetic gas exchange and chlorophyll fluorescence parameters. **(A)** Net photosynthetic rate; **(B)** Stomatal conductance; **(C)** Intercellular CO_2_ concentration; **(D)** Maximal efficiency of PSII photochemistry (Fv/Fm); **(E)** Actual efficacy of PSII (ΦPSII). Data are means ± SE calculated from three replicates. Different letters indicate a significant difference between the WT and both transgenic lines (ANOVA; *P* < 0.05). Three biological experiments were performed, which produced similar results.

We investigated the effects of drought stress on the photosynthetic capacity of the transgenic lines and WT. Four weeks old WT and transgenic tobacco plants were drought-stressed for 7 days, and then the foliar photosynthetic gas exchange parameters were measured as shown in **Figures 6A–C**. Under control conditions, there was no significant difference in leaf net photosynthetic rate (*Pn*) between transgenic plants and WT (**Figure 6A**). After 7-day drought stress, however, the decrease in WT was greater (24% reduction for WT) than that observed in both transgenic lines (5% reduction on average). Relative changes in stomatal conductance (*Gs*) were similar to the changes seen in *Pn* after drought stress, with the same trend as in *Pn* but to a smaller extent (**Figure 6B**). However, the wild-type was higher than both transgenic plants in intercellular CO_2_ concentration (*Ci*); showing an inverse relationship to *Gs* and *Pn* (**Figure 6C**).

Two measures of PSII photochemistry showed that no significant differences in F*v*/Fm and ΦPSII were observed between the wil-type and transgenic plants under normal conditions (**Figures 6D,E**). After drought stress for 7 day, The values of F*v*/Fm and ΦPSII decreased significantly in all types of plants. However, they were higher in both transgenic lines than the wild-type under drought conditions (**Figures 6D,E**); indicating that the PSII complex in transgenic plants could better endure stress-induced inactivation than the wild-type during drought stress. Together, these results indicate that photosynthesis damage in the transgenic plants was alleviated due to the over-expression of the *psbA*.

### Overexpression of *ZmpsbA* Altered Expression of Stress Defense Genes Under Drought Stress

**FIGURE 7:**
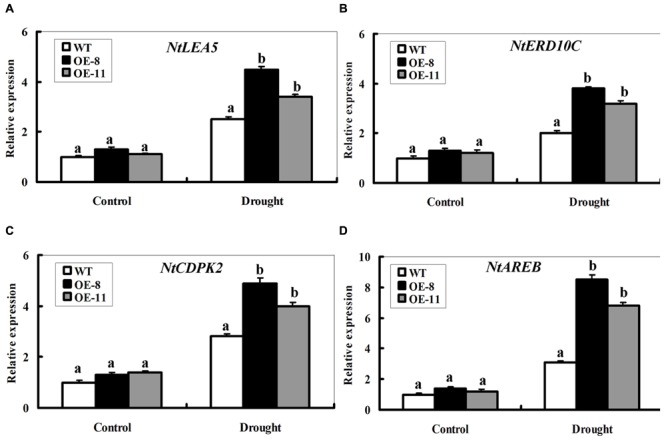
**Expression profiles of four stress-responsive genes in WT and *ZmpsbA*- transgenic lines during drought stress.** RNA was extracted from leaves sampled after 7 days of well-watering and drought stress and reverse-transcribed to synthesize cDNA, which was used for quantitative real-time PCR analysis with primers specific for four stress-responsive genes *NtLEA5* **(A)**, *NtERD10C* **(B)**, *NtCDPK2* **(C)**, and *NtAREB* **(D)**. mRNA levels of these genes were normalized to the transcripts of *Actin2* in the same samples. For each assay, the expression level of WT under control conditions was taken as 1.0, and data represented mean ± SE of three biological replicates. Bar indicates SE. Significant differences between wild-type and OE lines are indicated by different letters (ANOVA; *P* < 0.05).

To understand the effect of *ZmpsbA* OE, transcript levels of four representative stress defense genes were monitored under normal and drought conditions. These genes include two functional genes *NtLEA5* (late embryogenesis abundant 5) and *NtERD10C* (early responsive to dehydration 10C) and two regulatory genes *NtCDPK2* (calcium-dependent protein kinase 2) and *NtAREB* (ABA responsive element-binding protein, a bZIP transcription factor), which or whose homologues in other plant species have been shown to be functioning to alleviate abiotic stress (Kang et al., 2002; Kasuga et al., 2004; Witte et al., 2010). Real time PCR was used to examine transcript levels of the four genes between WT and both OE lines under normal and 7-day-drought conditions (**Figure 7**). Under normal conditions, the transcript level of each gene showed no significant changes between WT and OE plants. After the 7-day drought treatment, the transcript levels of the four genes were significantly elevated in both OE lines (increased by 2.5∼8 folds on average), while only induced slightly in the WT (increased by 0.8∼1.6 folds) (**Figures 7A–D**). Furthermore, it is noticeable that mRNA levels of the *NtAREB* in both OE lines were significantly higher than the WT (**Figure 7D**). These data indicated that *ZmpsbA* confers drought tolerance in tobacco possibly through regulating expression of these stress defense genes.

## Discussion

D1 protein plays a pivotal role in protecting PSII from oxidative damage during environmental stresses (Rintämaki et al., 1996). Our genetic evidence suggests that *ZmpsbA* confers drought tolerance by possibly regulating antioxidant system, photosynthetic capability, and stress defense gene expression in tobacco.

### Over-Expression of the *ZmpsbA* Improved Photosynthesis Capability in the Transgenic Plants Under Drought Stress

Drought stress causes damage to photosynthetic apparatus, which leads to photosynthesis inhibition, preceding function impairments of other cells. Usually, variations in photosynthetic parameters under drought stress are good indicators of drought tolerance for plants (Chaves et al., 2009; Osakabe et al., 2014). As shown in **Figures 6A–C**, the *Pn* and *Gs* of the WT and OE plants decreased after 7-day drought stress. Especially, the *Gs* of both OE lines has less reductions than that of the WT after drought (**Figure 6B**). This change trend in *Gs* is similar to that in *Pn* between WT and OE lines, indicating that the higher *Pn* in OE lines was mainly dependent on *Gs* regulation. However, the changes observed in *Ci* (**Figure 6C**) showed an inverse relationship to *Gs* and *Pn*, that is, the value of *Ci* in the wild-type was higher than both transgenic lines. This was caused possibly by the differential decreases of *Pn* between the wild-type and transgenic plants (**Figure 6A**). Over-expression of *ZmpsbA* enhanced protection in biochemical components of photosynthesis, which might result in higher CO_2_ assimilation and lower *Ci* in these transgenic plants than the wild type plants. This suggests that over-expression of the *ZmpsbA* ameliorates photosynthesis inhibition under drought stress. This result is in agreement with a previous report that over-expression of the *psbA* gene confers tolerance to photoinhibition of PSII in *synechococcus* cells (Soitamo et al., 1996).

Chloroplasts are cell compartments highly vulnerable to oxidative stress caused by drought stress and the D1 protein is the primary target of the damage. To protect PSII from complete inactivation and disassembly, the D1 protein requires high efficient turnover (Adir et al., 2003). The increased tolerance to drought stress in *ZmpsbA* OE lines might be attributed to high efficient turnover of D1 protein in the PSII repair cycle directly during the stress. These results plus previous reports indicate that *psbA* might play pivotal roles in maintaining stable photosynthesis capability by protecting PSII from oxidative damage during abiotic stress.

### *ZmpsbA* is Involved in Drought Tolerance Possibly Through Regulating Several Stress Defense Gene Expression

The transcript levels of four stress defense genes (*NtLEA5, NtERD10C, NtAREB*, and *NtCDPK2*) in the OE lines had much higher increases than those in the WT during drought stress (**Figure 7**). *LEA5* and *ERD10C* have pivotal roles in withstanding cellular dehydration; high expression of both genes can provide more chaperones or protective proteins for maintaining membrane integrity to sustain plant growth during drought (Hundertmark and Kincha, 2008; Kovacs et al., 2008). *CDPK2* and *AREB* are important regulatory genes, which participate in stress signal transduction during drought conditions (Shinozaki and Yamaguchi-Shinozaki, 2007; Saibo et al., 2009; Witte et al., 2010). In addition, compared to other three genes, the bZIP transcription factor gene *NtAREB* in the OE transgenic lines was induced to higher level than WT (**Figure 7D**). However, the question on how the chloroplast-localized D1 protein affects these stress-responsive gene transcription is elusive. Fortunately, some reports has shown that photosynthetic redox controls nuclear gene expression by plastid-to-nucleus retrograde signaling (Fey et al., 2005; Woodson and Chory, 2008; Kakizaki et al., 2009). From our result plus previous findings, it can be presumed that ZmpsbA may affect stress defense gene expression through modulating the redox state of photosynthetic electron transport chain components in plastid-to-nucleus signaling during drought stress.

### The Antioxidant Machinery is Involved in *ZmpsbA*-Mediated Drought Stress Tolerance

Reactive oxygen species accumulation induced by drought leads to cell toxicity, membrane peroxidation and even cell death (Apel and Hirt, 2004; Nishiyama et al., 2006). The antioxidant enzymes have pivotal functions in protecting plants from ROS-induced oxidative damage (Apel and Hirt, 2004). We compared the differences in the activities of antioxidant enzymes, ROS accumulation and lipid peroxidation between the wild-type and transgenic plants under drought stress, and found that over-expression of *ZmpsbA* displayed higher activities of antioxidant enzymes SOD, CAT, and POD and decreased ROS accumulation compared with the WT during drought stress (**Figures 5** and **6**); implying the roles of the over-expressed D1 in eliminating excessive ROS and enhancing antioxidant ability. It is well known that plants with high levels of these antioxidant enzymes show tolerant to drought, salinity or oxidative stress (Noreen and Ashraf, 2009; Wang et al., 2009; Kim et al., 2010). More recently, a conjoint analysis of transcriptome and proteome on tobacco *psbA* deletion mutants has revealed that expression of several antioxidant enzymes such as CAT and POD significantly decreased, indicating that the normal redox state of chloroplastsis was disrupted in the *psbA* deletion mutants (Leelavathi et al., 2011). Thus, it could be presumed that ZmpsbA may regulate activities of antioxidant enzymes through modulating the redox state of photosynthetic system or photosynthetic by-products such as ROS during drought stress. In summary, our genetic and physiological evidence has demonstrated that *ZmpsbA* confers drought stress tolerance by enhancing activities of antioxidant enzymes that scavenge excessive ROS and reduce membrane injury indirectly. However, the exact mechanism by which *psbA* affects ROS metabolism during drought are unclear. Further work will be needed to identify ROS metabolism-related genes regulated by *psbA* during drought stress through transcriptome analysis using the *psbA* over-expression lines.

## Author Contributions

ZX designed the research. YH, MW, YW, and ZX performed research and conducted data analyses. ZX wrote the manuscript.

## Conflict of Interest Statement

The authors declare that the research was conducted in the absence of any commercial or financial relationships that could be construed as a potential conflict of interest.

## Supplementary Material

Figure S1 **Sequence alignment of *psbA* cDNA between maize and tobacco**. The red long horizontal arrows designate the primers used for semiquantitative RT-PCR.

## Abbreviations

Φ_PSII_: actual efficiency of PSII
C*i*: intercellular CO_2_ concentration
*F_v_/F_m_*: maximal efficiency of PSII photochemistry
G*s*: stomatal conductance
MDA: malondialdehyde
P*n*: net photosynthesis rate
PSII: Photosystem II
