# Supplementary material for: Overexpression of the Maize psbA Gene Enhances Drought Tolerance Through Regulating Antioxidant System, Photosynthetic Capability, and Stress Defense Gene Expression in Tobacco
Source: Front Plant Sci. 2016 Jan 12;6:1223. doi: 10.3389/fpls.2015.01223 (PMC4709446; doi:10.3389/fpls.2015.01223)
Supplement: Supplementary file 1 [file Table_1.DOC]

**Supplementary data**

**Table S1 PCR primers used in this study**

Note: Basic residues are indicated in bold.

| Primer name | Primer sequence | Use |
| --- | --- | --- |
| ZmpsbA-sqF1  ZmpsbA-sqR1  NtLEA-QF  NtLEA-QR  NtERD10C-QF  NtERD10C-QR  NtCDPK2-QF  NtCDPK2-QR  NtAREB-QF  NtAREB-QR  NtActin2-QF  NtActin2-QR | CAATTTTAGAGAGACGCGAAAGTAC  GTAAAAATGC AATCCGATCG CC  5'-TTGTTAGCAGGCGTGGGTAT-3'  5'-CTCTCGCTCTTGTTGGGTTC-3'  5'-ACGGACGAATACGGCAATC-3'  5'-TCTCCTTAATCTTCTCCTTCATCC-3'  5'- AGGTGAGCTTTTCGATAGGATTATT-3'  5'- ACTTCTGGTGCAACATA GTAAGGAC-3'  5'-TCTTCACAGCAAAAGCCTCA-3'  5'-GTGACCCCATTATGCAATCC-3'  5'-TGGCATCACACTTTCTACAA -3'  5'-CAACGGAATCTCTCAGCTCC-3' | Semi-qRT-PCR for maize psbA expression in transgenic tobacco  qRT-PCR for tobacco *LEA* expression  qRT-PCR for tobacco *ERD10C* expression  qRT-PCR for tobacco *CDPK2* expression  qRT-PCR for tobacco *AREB* expression  qRT-PCR for tobacco *Actin2* expression |
