# Supplementary figures and images for: Overexpression of the Maize psbA Gene Enhances Drought Tolerance Through Regulating Antioxidant System, Photosynthetic Capability, and Stress Defense Gene Expression in Tobacco
Source: Front Plant Sci. 2016 Jan 12;6:1223. doi: 10.3389/fpls.2015.01223 (PMC4709446; doi:10.3389/fpls.2015.01223)

## Slide 1
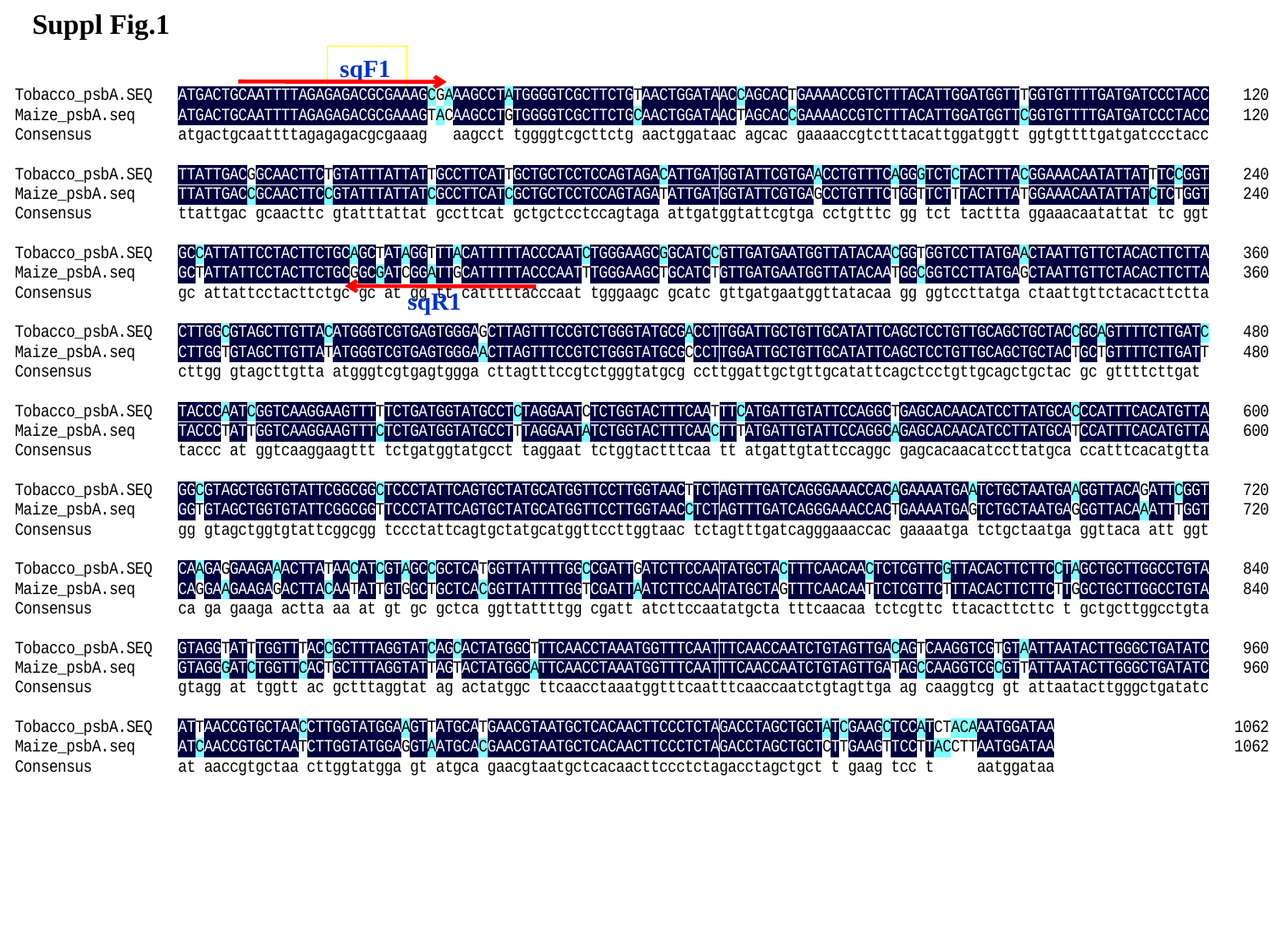

Suppl Fig.1
sqF1
sqR1

Supplement: Figure S1 — Sequence alignment of psbA cDNA between maize and tobacco. The red long horizontal arrows designate the primers used for semiquantitative RT-PCR. [file Presentation_1.PPT]
